# *Babesia* Species Detected in Deer from Southwest England

**DOI:** 10.3390/pathogens14040303

**Published:** 2025-03-22

**Authors:** Hope Leverett, Ternenge T. Apaa, Harriet McFadzean, Nicholas Johnson

**Affiliations:** 1 One Virology—Wolfson Centre for Global Viral Research, School of Veterinary Medicine and Science, University of Nottingham, Loughborough LE12 5RD, UK; 2 Vector Borne Diseases, Virology Department, Animal and Plant Health Agency, Woodham Lane, Addlestone KT15 3NB, UK; ter.apaa@apha.gov.uk; 3 Animal and Plant Health Agency, Starcross Veterinary Investigation Centre, Staplake Mount, Starcross EX6 8PE, UK; harriet.mcfadzean@apha.gov.uk; 4 Faculty of Health and Medical Sciences, University of Surrey, Guildford GU2 7XH, UK

**Keywords:** red deer, fallow deer, *Babesia*

## Abstract

*Babesia* species have been detected in deer across Europe, and deer grazing in the same location as livestock may increase the risk of transmission of species such as the parasite *B. divergens.* Bovine babesiosis and the cost of treatment increase the economic burden on farmers. To determine the presence of *Babesia* species in wild deer populations in the counties of Devon and Somerset, Southwest England, blood samples were collected from red (*Cervus elaphus*) and fallow (*Dama dama*) deer as part of routine deer management during late 2022 and early 2023. Extracted DNA samples were tested for the presence of piroplasm DNA by polymerase chain reaction. Amplicons were sequenced to identify the species present in samples based on single-nucleotide polymorphisms within the 18S rRNA gene. Two species of *Babesia* were detected: a *B. divergens/capreoli* species detected in both red and fallow deer and a *Babesia* species related to *B. odocoilei* in a single fallow deer, a species that has been detected in deer across Great Britain. The presence of *B. divergens/capreoli* in deer blood from these areas provides evidence that wild deer could serve as a reservoir for this parasite within Southern England.

## 1. Introduction

Babesiosis is a disease affecting livestock and wildlife species. Clinical signs of babesiosis vary depending on host age, parasite load, immunity to babesiosis and *Babesia* species. In deer, infection is usually asymptomatic, and disease is rare, although acute disease with clinical signs such as fever, lethargy, anorexia and anaemia characterised by jaundice (haemolytic) and petechiae (thrombocytopaenia) have been reported [1]. There are a few fatal cases recorded in wild deer, mostly in roe deer [2], captive/semi-captive reindeer [3,4] and chamois [5]. Through comprehensive field surveys in mainland Europe, the ensemble of *Babesia* species infecting wild deer has been established, including *B. divergens*, *B. capreoli*, *Babesia* sp. EU1 (commonly referred to as *B. venatorum*) and *B*. cf. *odocoilei* [6,7,8,9]. In some surveys, *B. motasi* has also been detected [10]. Some *Babesia* species, including *B. divergens* and *B. venatorum,* are zoonotic in Europe [11], with infection occurring in immunosuppressed or splenectomised patients [12].

Great Britain has six species of deer: two indigenous species, red (*Cervus elaphus*) and roe (*Capreolus capreoli*); fallow deer (*Dama dama*) were introduced in medieval times, and three have been introduced over the past 150 years: sika (*Cervus nippon*), Reeve’s muntjac (*Muntiacus reevesi*) and Chinese water deer (*Hydropotes inermis*). Each has a different geographical range [13], and all are considered to be expanding in distribution [14]. *Babesia* species have been identified in red deer in Ireland [15], Scotland [16] and England [17], with two of these studies [15,16] suggesting the presence of *B. divergens* in red deer. However, some authors [18] have cast doubt on this identification, and the question remains as to whether the strain of *B. divergens* detected in wild ungulates is the same as that present in livestock and occasional cases of human babesiosis [19]. To investigate the potential for deer to act as a reservoir for *Babesia* spp. in Southwest England, an area of high livestock density, high tick abundance and prevalent tick-borne disease [20], this study surveyed deer samples from two species for *Babesia* spp. collected from the counties of Devon and Somerset.

## 2. Materials and Methods

### 2.1. Study Area and Samples

The study area is a combination of moorland, pasture and woodland spanning the counties of Devon and Somerset in Southwest England. The dominant deer population is the red deer (*Cervus elaphus*), with numbers estimated to be over 3000. Other species present include fallow (*Dama dama*) and roe (*Capreolus capreolus*). The study area is also used to graze cattle, horses and sheep. Routine deer management control occurs annually during the late autumn and winter months to control numbers. A volume of 5–10 mL of coagulated blood was collected from 76 deer carcasses provided to the Animal and Plant Health Agency (APHA) by local deer managers. Data on the age, sex and species was collected. The age of the host animal was divided into two categories: juvenile and adult. For red deer, adults were classified as over five years in males and older than two years in females. For fallow deer, the criterion was older than two years in males and over one year in females.

### 2.2. DNA Extraction, Pan-Piroplasm PCR and Sequencing

DNA was extracted from blood samples using the DNeasy Blood and Tissue kit (QIAgen, Manchester, UK) following the manufacturer’s protocol and stored at 4 °C until further processing. Samples were tested for the presence of *Babesia* species using a published Pan-piroplasm 18S rRNA gene PCR assay using primers Piro A (5′- AATACCCAATCCTGACACAGGG-3′) and Piro B (5′-TTAAATACGAATGCCCCCAAC-3′) [21,22], which amplifies a 423 base-pair (bp) fragment. The PCR reaction consisted of 2x iTaq Universal Sybr mix (QIAgen, Manchester, UK), 0.4 μM of both forward and reverse primers and nuclease-free water in a total volume of 23 μL. A 2 μL volume of eluted DNA for each sample was added per reaction. PCR thermal cycling conditions consisted of denaturation at 94 °C for 5 min followed by 45 cycles of 94 °C for 30 s, 60 °C for 30 s, 72 °C for 60 s and a final extension at 72 °C for 7 min. The products were examined by gel electrophoresis on a 1.5% agarose gel stained with Sybr Safe© (Thermo Fisher Scientific, Crawley, UK and visualised with UV illumination. Positive samples were sequenced as described previously [22].

### 2.3. DNA Sequence Editing, Assembly and Phylogenetic Analysis

Sequence quality assessment, editing and trimming of primers, alignment and generation of consensus for each sample were achieved using Seqman Pro and Editseq programmes in DNASTAR Lasergene v15. *Babesia* species sequences generated in this study were submitted to GenBank under the accession numbers (OQ884253 and OQ884261). The NCBI online BLASTn search was first carried out to demonstrate the percentage similarity between sequences obtained and the *Babesia* species sequence present in the NCBI database. Reference sequences with high-scoring BLASTn hits (AY046576, FJ944827, KF773722, MH697657 and KC2499443, MT550684, GQ304525, MT151379 and KY242380) and those submitted from published literature were downloaded. Multiple sequence (reference and those obtained from this study) alignment, calculation of the best fitting nucleotide substitution model (Tamura-Nei) and construction of maximum-likelihood phylogenetic tree at 1000 bootstrap approximations were carried out in MEGA 11. The *B. pecorum* sequence (GenBank accession number KC249943) was used to root the tree.

### 2.4. Statistical Analysis

The prevalence and 95% confidence intervals (CI) were calculated using Wilson’s method on the Epitools Website [23]. Statistical analyses were performed using Prism 9. Contingency table analysis using Fisher’s exact test was performed to test for associations of the prevalence of *Babesia* with host age and sex. A significance level of *p* = 0.05 was used.

## 3. Results and Discussion

*Babesia* spp. were detected in 5 of 38 red deer samples (13.2%; CI 95% (5.75–26.33)) and 4 of 38 fallow deer (10.5%; CI 95% (4.17–24.1) (Table 1). Six of the *Babesia* species sequences obtained from red and fallow deer showed high sequence identity (>99%) with a *B. divergens* sequence (MT550684) obtained from cattle from the county of Dorset in 2019. Phylogenetic analysis confirmed the presence of at least two *Babesia* species within the deer populations of the study area (Figure S1). However, this analysis could not entirely discriminate between *B. divergens* and *B. capreoli*. The second sequence detected from a female fallow deer was similar but genetically distinct to *B. odocoilei*. Statistical testing showed no significant difference between positive cases of *Babesia* and sex or age for both species (*p* > 0.05).

Surveillance data collected between 2018 and 2022 by APHA indicate that cases of bovine babesiosis are reported in discrete areas of Great Britain, including the Highlands and southwest areas of Scotland, Northern England, Central Wales and counties of Southwest England (Figure S2). This latter area, comprising the counties of Devon, Cornwall and Somerset, is considered a particular “hotspot” for tick-borne diseases such as bovine babesiosis [20]. However, sporadic outbreaks of bovine babesiosis are unpredictable, and the origin of most infections is never identified. This study demonstrates the presence of *B. divergens*/*B. capreoli* in deer species in Southwest England, supporting previous reports where deer species have been considered potential reservoirs for *Babesia* species. There have been a range of prevalence identified in other UK-based studies on red deer ranging from 4.8% (n = 105) in farmed red deer [17] to 18.3% (n = 60) across an area of Scotland [16]. Gray and co-workers reported a high prevalence of *B. divergens* of 46% (n = 24) in deer sampled from fields where cattle were also infected. That study also detected *B. odocoilei*-like *Babesia* in the deer population in Scotland. In previous studies from mainland Europe involving fallow deer, the prevalence was noted at 13.9% (n = 43) [24] and 14.3% (n = 7) [9]. The prevalence of *Babesia* species of 10.5% in fallow deer and 13.2% in red deer are therefore not unusual.

In Europe, *B. divergens* can be mistaken for *B. capreoli*, a species associated with infection in roe deer (*Capreolus capreolus*), due to morphological similarities in thin blood smears and high sequence identity within the 18S rRNA gene. There is limited nucleotide variation between the 18S rRNA sequences between these two species, although distinct nucleotide polymorphisms have been identified [2]. However, both species are clearly distinct from the second species, often described as *B. odocoilei*-like, detected in this and other studies (Table 2, Figure S3).

Both red and fallow deer are thought to be growing in number in Great Britain and Europe [25]. A recent survey carried out by the British Deer Society demonstrated the increase in distribution and comments on the increased abundance of deer [13]. Upland grazing of both cattle and several species of wild deer is common on moorland in Southwest England and forms an integral part of landscape management. Given that deer can travel large distances, particularly due to male dispersal behaviour [26], there is concern that they could facilitate the spread of *B. divergens* and/or *B. divergens*-infected ticks, potentially introducing the parasite to new areas and expose naïve cattle populations. However, it is also possible that transmission can occur in the opposite direction from livestock into the deer population. Further investigation is required to confirm the species infecting deer populations present in Southwest England and the potential for pathogen exchange between livestock and resident wild ungulates. There are several methods used to reduce the impact of bovine babesiosis infection, including pasture management and treatment of infected cattle with imidocarb dipropionate [27]. However, the former option is not always feasible in moorland habitats due to harsh environmental conditions and conservation guidelines set out in environmental stewardship agreements. Anti-parasitic treatment requires the withdrawal of milk and meat from entering the food chain for proscribed periods, adding to the economic cost of infection [28]. Pour-on treatments are currently only licenced for sheep in the United Kingdom but can be prescribed “under the cascade” to other species where clinically appropriate. However, there is concern that increasing acaricide applications could accelerate resistance in *I. ricinus* and limit control methods in the future [29]. While this treatment reduces the number of ticks present in cattle, tick feeding on alternative sources such as deer will continue. This may then lead to a loss of poorly understood immunity to *Babesia* and create naïve cattle populations that are susceptible to future infection.

## Figures and Tables

**Table 1:** Prevalence of *Babesia* detected in deer samples collected from Devon and Somerset.

Cervid Species	*Babesia* Positive/Total	Prevalence (95% CI)
Fallow deer (*Dama dama*)	4/38	10.5% (4.17–24.1)
Red deer (*Cervus elaphus*)	5/38	13.2% (5.75–26.33)

**Table 2:** Summary of sample and interpretation of 18S rDNA sequences generated in this study.

Sample ID	Date Sampled (month/year)	Description	18S rDNA Bases at Position 631/663	*Babesia* Species Detected *	GenBank Acc. No.
M0224	10/2022	Red deer/male	A/A	*B. divergens/capreoli*	OQ884256
M0365	10/2022	Red deer/male	A/A	*B. divergens/capreoli*	OQ884258
M0152	11/2022	Red deer/female	A/A	*B. divergens/capreoli*	OQ884259
M0346	02/2023	Red deer/**	A/A	*B. divergens/capreoli*	OQ884261
M0583	09/2022	Fallow deer/male	A/A	*B. divergens/capreoli*	OQ884254
M0247	11/2022	Fallow deer/female	A/A	*B. divergens/capreoli*	OQ884253
M0259	11/2022	Fallow deer/female	-	*B*. cf *odocoilei*	OQ884255

* Species determined by NCBI nucleotide BLAST search (https://blast.ncbi.nlm.nih.gov/Blast.cgi accessed on 10 February 2025) of partial (364 base pairs) 18S rDNA sequence and single-nucleotide polymorphisms suggested by the authors of [2]. ** Data not recorded.

## Data Availability

Sequence data are available in NCBI GenBank under accession numbers OQ884253—OQ884261).

## Supplementary Materials

The following supporting information can be downloaded at https://www.mdpi.com/article/10.3390/pathogens14040303/s1. Figure S1: Comparison of 18S rDNA sequences derived from deer samples originating in southwest England; Figure S2: Map showing the distribution of bovine babesiosis cases within Great Britain reported to APHA between 2018 and 2022; Figure S3: Schematic showing the sequence within the 18S rRNA gene that distinguishes *Babesia divergens* and *B. capreoli* based on Malandrin et al., 2010 [2].
