# Supplementary material for: Babesia Species Detected in Deer from Southwest England
Source: Pathogens. 2025 Mar 22;14(4):303. doi: 10.3390/pathogens14040303 (PMC12030073; doi:10.3390/pathogens14040303)
Supplement: Supplementary file 1 [file pathogens-14-00303-s001.zip › pathogens-3498921-supplementary.pdf]

Supplementary Figures

Figure S1. Comparison of 18S rDNA sequences derived from deer samples originating in southwest England. The phylogeny was constructed using the Maximum likelihood method with 1000 bootstrap approximations. The tree is rooted with *B. pecorum* as an outgroup and includes bootstrap values ≥ 95%.

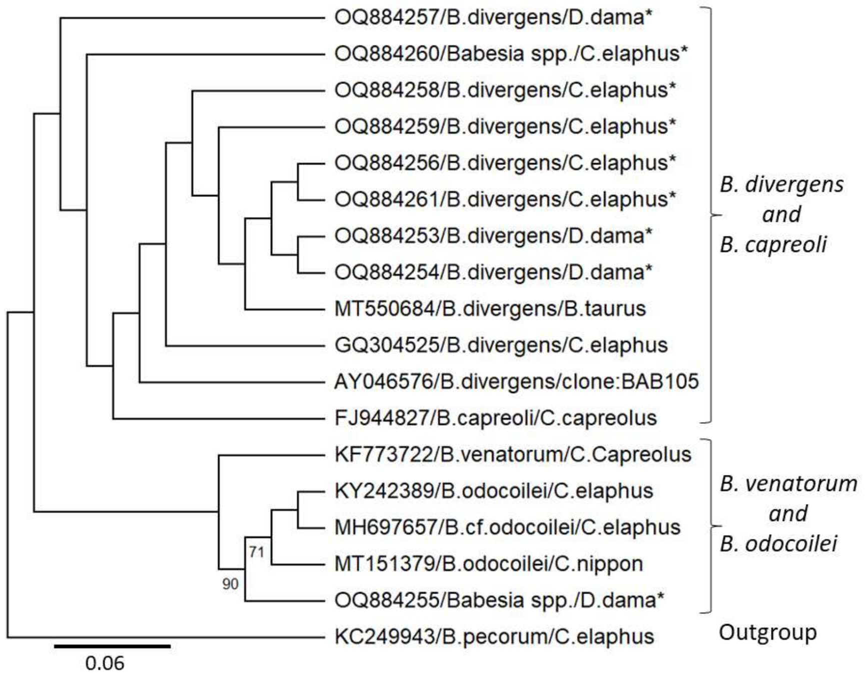

Figure S2. Map showing the distribution of bovine babesiosis cases within Great Britain reported to APHA between 2018 and 2022. The map was generated at APHA Vet Gateway: Livestock disease surveillance dashboards using software under licence from https://public.tableau.com. (https://public.tableau.com/app/profile/siu.apha/viz/CattleDashboard/Overview), accessed 19/06/2023.

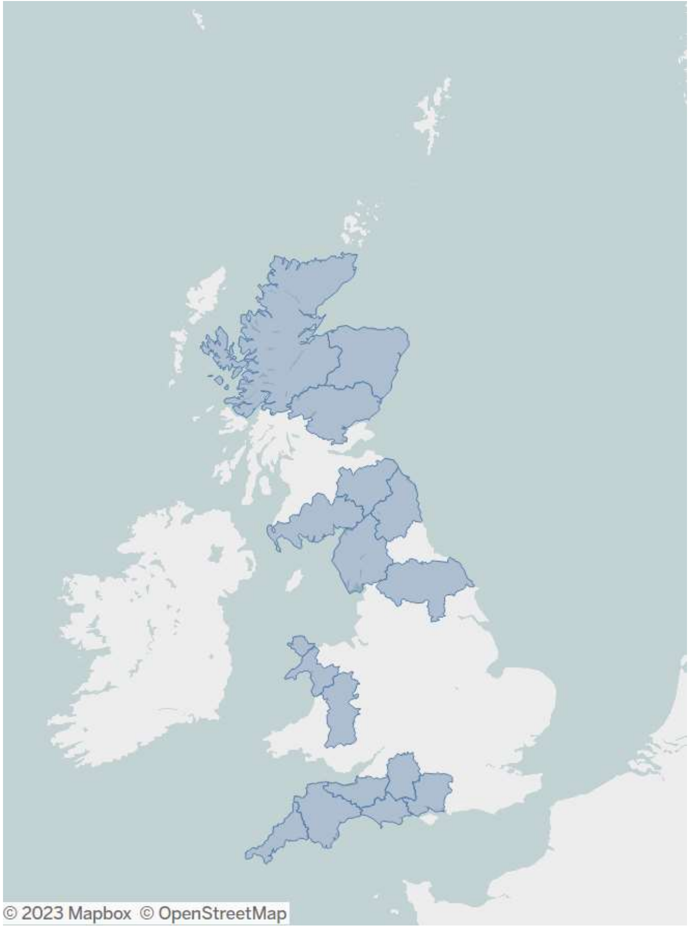

Figure S3. Schematic showing the sequence within the 18S rRNA gene that distinguishes *Babesia divergens* and *B. capreoli* based on Malandrin *et al.*, 2010 [2].

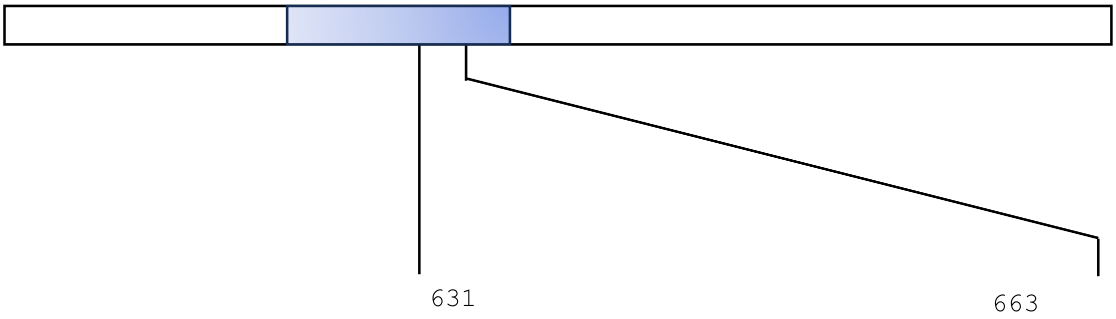


```
AY098643 B. capreoli  TGATGTCGAGATTGCACTTCGCTTTTGGGATTTTT
AY046576 B. divergens -A-------------------------------A-
OQ884256              -A-------------------------------A-
OQ884258              -A-------------------------------A-
OQ884249              -A-------------------------------A-
OQ884261              -A-------------------------------A-
OQ884254              -A-------------------------------A-
OQ884253              -A-------------------------------A-
OQ884254              -A-------------------------------A-
```
